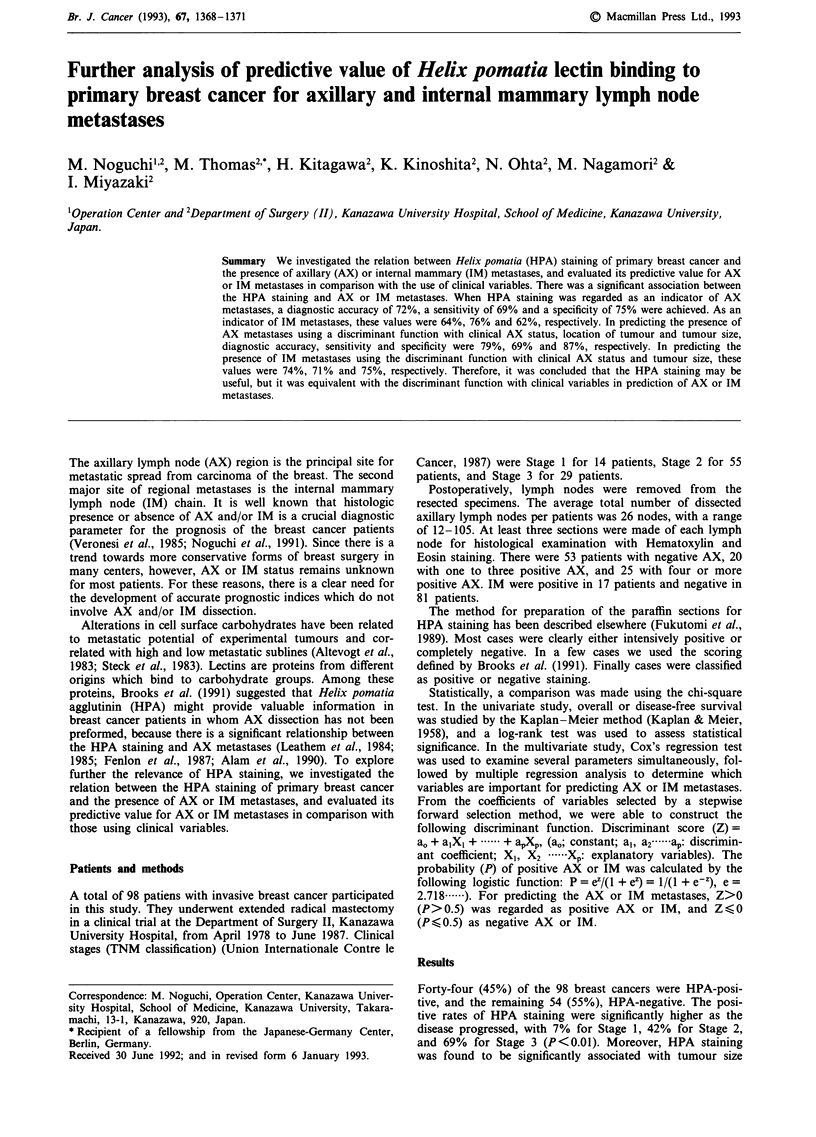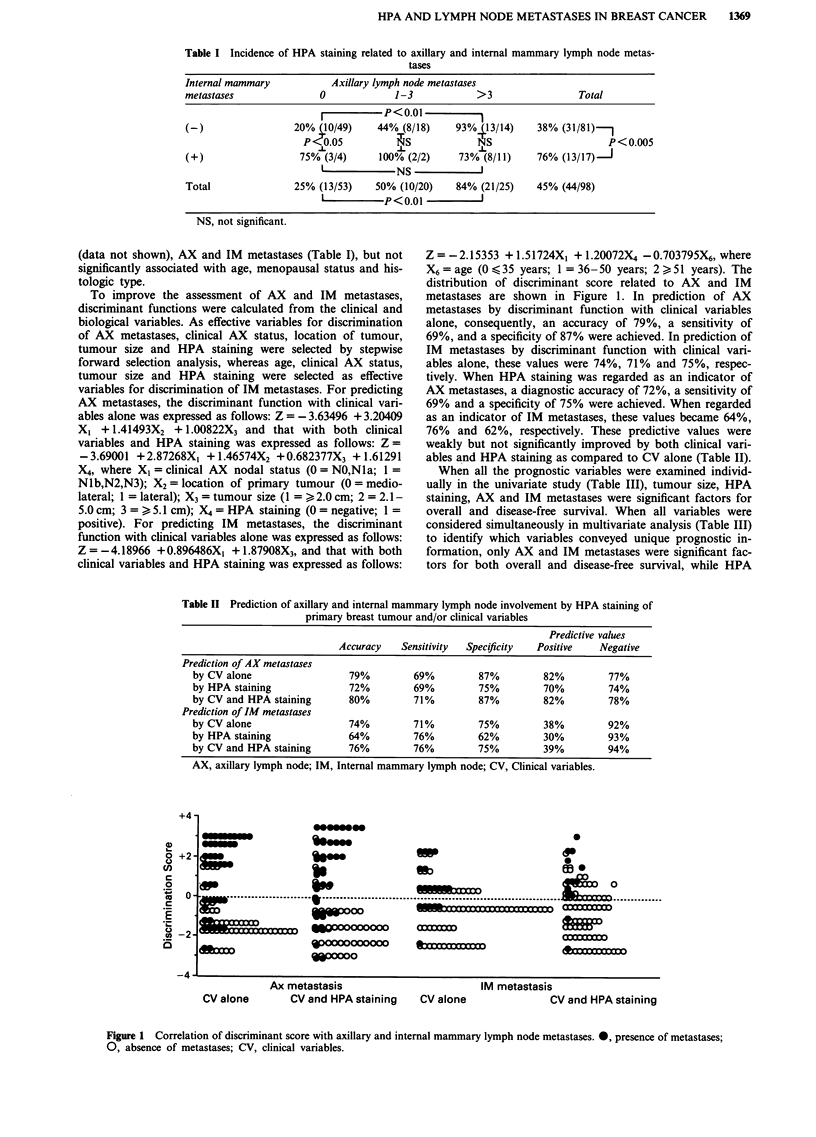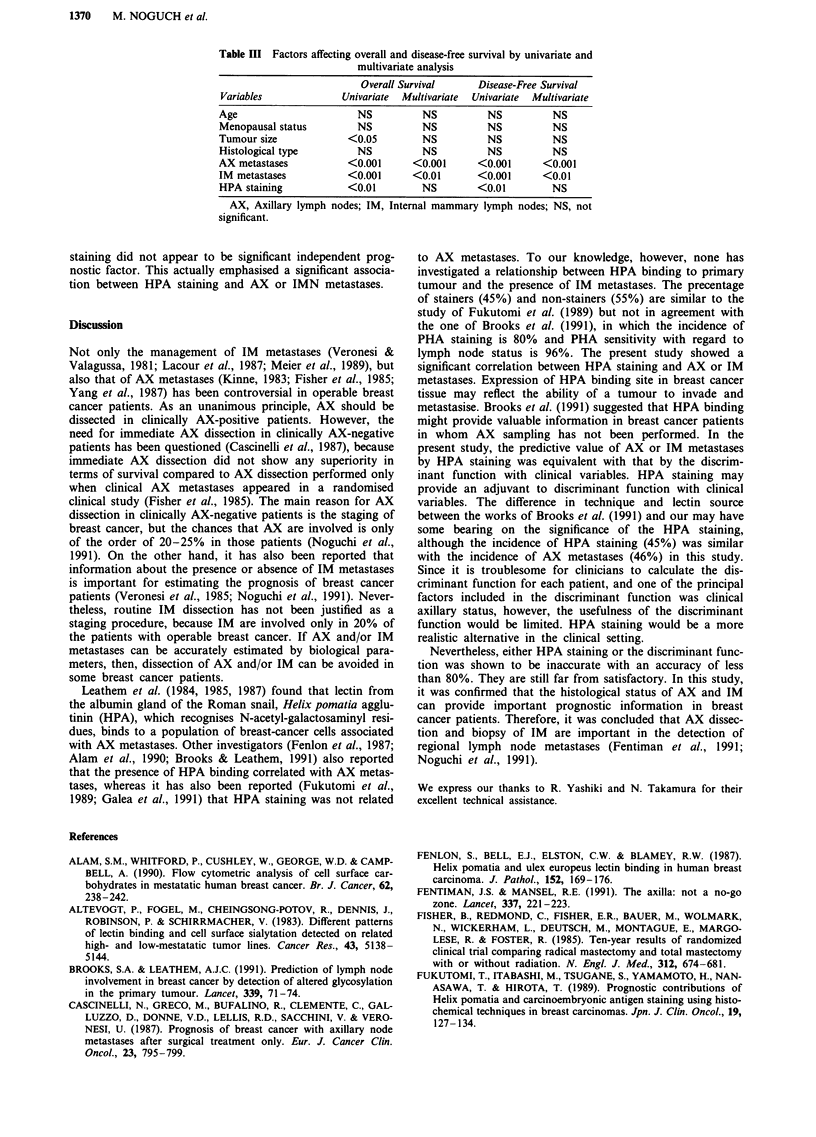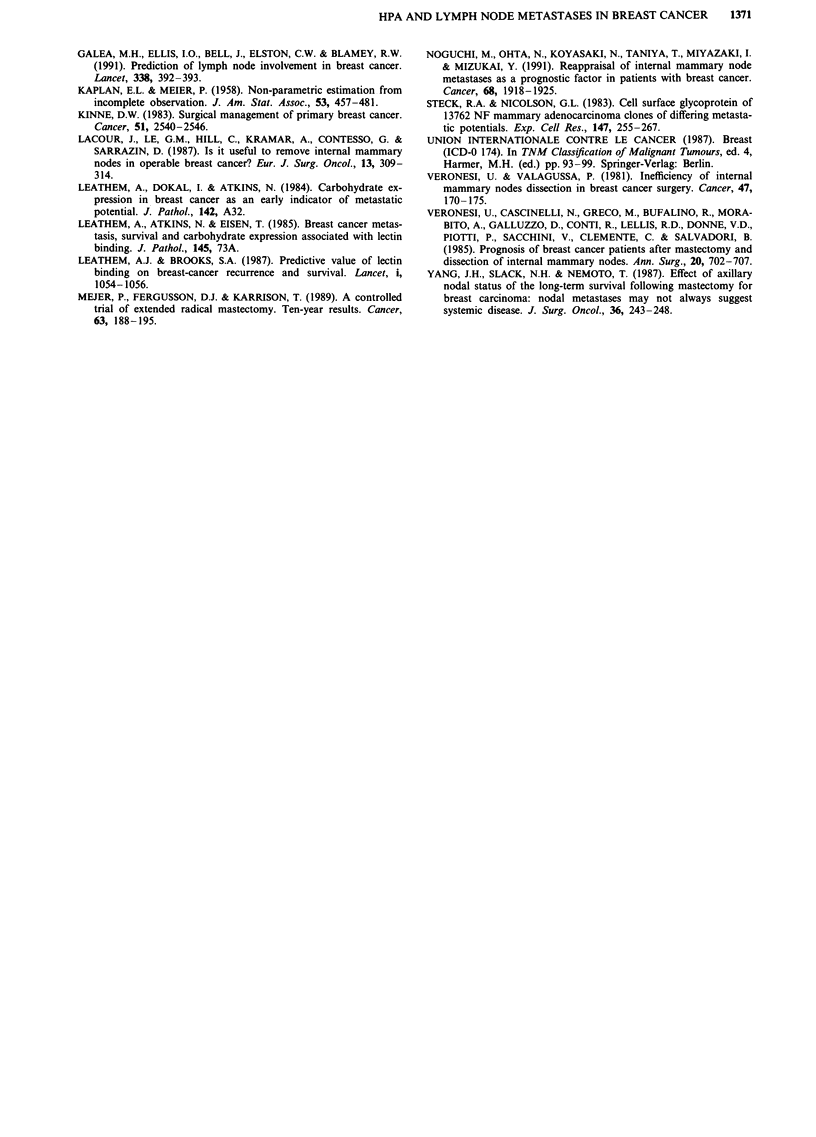# Further analysis of predictive value of Helix pomatia lectin binding to primary breast cancer for axillary and internal mammary lymph node metastases.

**DOI:** 10.1038/bjc.1993.253

**Published:** 1993-06

**Authors:** M. Noguchi, M. Thomas, H. Kitagawa, K. Kinoshita, N. Ohta, M. Nagamori, I. Miyazaki

**Affiliations:** Operation Center, Kanazawa University Hospital, School of Medicine, Japan.

## Abstract

We investigated the relation between Helix pomatia (HPA) staining of primary breast cancer and the presence of axillary (AX) or internal mammary (IM) metastases, and evaluated its predictive value for AX or IM metastases in comparison with the use of clinical variables. There was a significant association between the HPA staining and AX or IM metastases. When HPA staining was regarded as an indicator of AX metastases, a diagnostic accuracy of 72%, a sensitivity of 69% and a specificity of 75% were achieved. As an indicator of IM metastases, these values were 64%, 76% and 62%, respectively. In predicting the presence of AX metastases using a discriminant function with clinical AX status, location of tumour and tumour size, diagnostic accuracy, sensitivity and specificity were 79%, 69% and 87%, respectively. In predicting the presence of IM metastases using the discriminant function with clinical AX status and tumour size, these values were 74%, 71% and 75%, respectively. Therefore, it was concluded that the HPA staining may be useful, but it was equivalent with the discriminant function with clinical variables in prediction of AX or IM metastases.


Br. J. Cancer (1993), 67, 1368–1371 © Macmillan Press Ltd., 1993

### Further analysis of predictive value of *Helix pomatia* lectin binding to primary breast cancer for axillary and internal mammary lymph node metastases

M. Noguchi[1,2], M. Thomas[2,*], H. Kitagawa[2], K. Kinoshita[2], N. Ohta[2], M. Nagamori[2] & I. Miyazaki[2]

[1]*Operation Center and* [2]*Department of Surgery (II), Kanazawa University Hospital, School of Medicine, Kanazawa University, Japan.*

**Summary** We investigated the relation between *Helix pomatia* (HPA) staining of primary breast cancer and the presence of axillary (AX) or internal mammary (IM) metastases, and evaluated its predictive value for AX or IM metastases in comparison with the use of clinical variables. There was a significant association between the HPA staining and AX or IM metastases. When HPA staining was regarded as an indicator of AX metastases, a diagnostic accuracy of 72%, a sensitivity of 69% and a specificity of 75% were achieved. As an indicator of IM metastases, these values were 64%, 76% and 62%, respectively. In predicting the presence of AX metastases using a discriminant function with clinical AX status, location of tumour and tumour size, diagnostic accuracy, sensitivity and specificity were 79%, 69% and 87%, respectively. In predicting the presence of IM metastases using the discriminant function with clinical AX status and tumour size, these values were 74%, 71% and 75%, respectively. Therefore, it was concluded that the HPA staining may be useful, but it was equivalent with the discriminant function with clinical variables in prediction of AX or IM metastases.

The axillary lymph node (AX) region is the principal site for metastatic spread from carcinoma of the breast. The second major site of regional metastases is the internal mammary lymph node (IM) chain. It is well known that histologic presence or absence of AX and/or IM is a crucial diagnostic parameter for the prognosis of the breast cancer patients (Veronesi *et al*., 1985; Noguchi *et al*., 1991). Since there is a trend towards more conservative forms of breast surgery in many centers, however, AX or IM status remains unknown for most patients. For these reasons, there is a clear need for the development of accurate prognostic indices which do not involve AX and/or IM dissection.

Alterations in cell surface carbohydrates have been related to metastatic potential of experimental tumours and correlated with high and low metastatic sublines (Altevogt *et al*., 1983; Steck *et al*., 1983). Lectins are proteins from different origins which bind to carbohydrate groups. Among these proteins, Brooks *et al*. (1991) suggested that *Helix pomatia* agglutinin (HPA) might provide valuable information in breast cancer patients in whom AX dissection has not been preformed, because there is a significant relationship between the HPA staining and AX metastases (Leathem *et al*., 1984; 1985; Fenlon *et al*., 1987; Alam *et al*., 1990). To explore further the relevance of HPA staining, we investigated the relation between the HPA staining of primary breast cancer and the presence of AX or IM metastases, and evaluated its predictive value for AX or IM metastases in comparison with those using clinical variables.

### Patients and methods

A total of 98 patiens with invasive breast cancer participated in this study. They underwent extended radical mastectomy in a clinical trial at the Department of Surgery II, Kanazawa University Hospital, from April 1978 to June 1987. Clinical stages (TNM classification) (Union Internationale Contre le Cancer, 1987) were Stage 1 for 14 patients, Stage 2 for 55 patients, and Stage 3 for 29 patients.

Postoperatively, lymph nodes were removed from the resected specimens. The average total number of dissected axillary lymph nodes per patients was 26 nodes, with a range of 12–105. At least three sections were made of each lymph node for histological examination with Hematoxylin and Eosin staining. There were 53 patients with negative AX, 20 with one to three positive AX, and 25 with four or more positive AX. IM were positive in 17 patients and negative in 81 patients.

The method for preparation of the paraffin sections for HPA staining has been described elsewhere (Fukutomi *et al*., 1989). Most cases were clearly either intensively positive or completely negative. In a few cases we used the scoring defined by Brooks *et al*. (1991). Finally cases were classified as positive or negative staining.

Statistically, a comparison was made using the chi-square test. In the univariate study, overall or disease-free survival was studied by the Kaplan–Meier method (Kaplan & Meier, 1958), and a log-rank test was used to assess statistical significance. In the multivariate study, Cox's regression test was used to examine several parameters simultaneously, followed by multiple regression analysis to determine which variables are important for predicting AX or IM metastases. From the coefficients of variables selected by a stepwise forward selection method, we were able to construct the following discriminant function. Discriminant score (Z) = $a_o + a_1X_1 + \cdots\cdots + a_pX_p$, ($a_o$; constant; $a_1$, $a_2 \cdots\cdots a_p$: discriminant coefficient; $X_1$, $X_2$ $\cdots\cdots X_p$: explanatory variables). The probability (*P*) of positive AX or IM was calculated by the following logistic function: $P = e^z/(1 + e^z) = 1/(1 + e^{-z})$, e = 2.718$\cdots\cdots$). For predicting the AX or IM metastases, $Z > 0$ ($P > 0.5$) was regarded as positive AX or IM, and $Z \leqslant 0$ ($P \leqslant 0.5$) as negative AX or IM.

### Results

Forty-four (45%) of the 98 breast cancers were HPA-positive, and the remaining 54 (55%), HPA-negative. The positive rates of HPA staining were significantly higher as the disease progressed, with 7% for Stage 1, 42% for Stage 2, and 69% for Stage 3 ($P < 0.01$). Moreover, HPA staining was found to be significantly associated with tumour size

Correspondence: M. Noguchi, Operation Center, Kanazawa University Hospital, School of Medicine, Kanazawa University, Takaramachi, 13-1, Kanazawa, 920, Japan.

* Recipient of a fellowship from the Japanese-Germany Center, Berlin, Germany.

Received 30 June 1992; and in revised form 6 January 1993.

**Table I** Incidence of HPA staining related to axillary and internal mammary lymph node metastases

| *Internal mammary metastases* | *Axillary lymph node metastases* 0 | *1–3* | *>3* | *Total* |
|---|---|---|---|---|
| | *P*<0.01 (0 vs >3) | | | |
| (–) | 20% (10/49) | 44% (8/18) | 93% (13/14) | 38% (31/81) |
| | *P*<0.05 | NS | NS | *P*<0.005 |
| (+) | 75% (3/4) | 100% (2/2) | 73% (8/11) | 76% (13/17) |
| | NS (0 vs >3) | | | |
| Total | 25% (13/53) | 50% (10/20) | 84% (21/25) | 45% (44/98) |
| | *P*<0.01 (0 vs >3) | | | |

NS, not significant.

(data not shown), AX and IM metastases (Table I), but not significantly associated with age, menopausal status and histologic type.

To improve the assessment of AX and IM metastases, discriminant functions were calculated from the clinical and biological variables. As effective variables for discrimination of AX metastases, clinical AX status, location of tumour, tumour size and HPA staining were selected by stepwise forward selection analysis, whereas age, clinical AX status, tumour size and HPA staining were selected as effective variables for discrimination of IM metastases. For predicting AX metastases, the discriminant function with clinical variables alone was expressed as follows: $Z = -3.63496 + 3.20409 X_1 + 1.41493X_2 + 1.00822X_3$ and that with both clinical variables and HPA staining was expressed as follows: $Z = -3.69001 + 2.87268X_1 + 1.46574X_2 + 0.682377X_3 + 1.61291 X_4$, where $X_1$ = clinical AX nodal status (0 = N0,N1a; 1 = N1b,N2,N3); $X_2$ = location of primary tumour (0 = mediolateral; 1 = lateral); $X_3$ = tumour size (1 = ⩾2.0 cm; 2 = 2.1–5.0 cm; 3 = ⩾5.1 cm); $X_4$ = HPA staining (0 = negative; 1 = positive). For predicting IM metastases, the discriminant function with clinical variables alone was expressed as follows: $Z = -4.18966 + 0.896486X_1 + 1.87908X_3$, and that with both clinical variables and HPA staining was expressed as follows: $Z = -2.15353 + 1.51724X_1 + 1.20072X_4 - 0.703795X_6$, where $X_6$ = age (0 ⩽35 years; 1 = 36–50 years; 2 ⩾51 years). The distribution of discriminant score related to AX and IM metastases are shown in Figure 1. In prediction of AX metastases by discriminant function with clinical variables alone, consequently, an accuracy of 79%, a sensitivity of 69%, and a specificity of 87% were achieved. In prediction of IM metastases by discriminant function with clinical variables alone, these values were 74%, 71% and 75%, respectively. When HPA staining was regarded as an indicator of AX metastases, a diagnostic accuracy of 72%, a sensitivity of 69% and a specificity of 75% were achieved. When regarded as an indicator of IM metastases, these values became 64%, 76% and 62%, respectively. These predictive values were weakly but not significantly improved by both clinical variables and HPA staining as compared to CV alone (Table II).

When all the prognostic variables were examined individually in the univariate study (Table III), tumour size, HPA staining, AX and IM metastases were significant factors for overall and disease-free survival. When all variables were considered simultaneously in multivariate analysis (Table III) to identify which variables conveyed unique prognostic information, only AX and IM metastases were significant factors for both overall and disease-free survival, while HPA

**Table II** Prediction of axillary and internal mammary lymph node involvement by HPA staining of primary breast tumour and/or clinical variables

| | *Accuracy* | *Sensitivity* | *Specificity* | *Predictive values Positive* | *Negative* |
|---|---|---|---|---|---|
| *Prediction of AX metastases* | | | | | |
| by CV alone | 79% | 69% | 87% | 82% | 77% |
| by HPA staining | 72% | 69% | 75% | 70% | 74% |
| by CV and HPA staining | 80% | 71% | 87% | 82% | 78% |
| *Prediction of IM metastases* | | | | | |
| by CV alone | 74% | 71% | 75% | 38% | 92% |
| by HPA staining | 64% | 76% | 62% | 30% | 93% |
| by CV and HPA staining | 76% | 76% | 75% | 39% | 94% |

AX, axillary lymph node; IM, Internal mammary lymph node; CV, Clinical variables.

**Figure 1** Correlation of discriminant score with axillary and internal mammary lymph node metastases. ●, presence of metastases; O, absence of metastases; CV, clinical variables.

**Table III** Factors affecting overall and disease-free survival by univariate and multivariate analysis

| Variables | Overall Survival Univariate | Overall Survival Multivariate | Disease-Free Survival Univariate | Disease-Free Survival Multivariate |
|---|---|---|---|---|
| Age | NS | NS | NS | NS |
| Menopausal status | NS | NS | NS | NS |
| Tumour size | <0.05 | NS | NS | NS |
| Histological type | NS | NS | NS | NS |
| AX metastases | <0.001 | <0.001 | <0.001 | <0.001 |
| IM metastases | <0.001 | <0.01 | <0.001 | <0.01 |
| HPA staining | <0.01 | NS | <0.01 | NS |

AX, Axillary lymph nodes; IM, Internal mammary lymph nodes; NS, not significant.

staining did not appear to be significant independent prognostic factor. This actually emphasised a significant association between HPA staining and AX or IMN metastases.

### Discussion

Not only the management of IM metastases (Veronesi & Valagussa, 1981; Lacour *et al.*, 1987; Meier *et al.*, 1989), but also that of AX metastases (Kinne, 1983; Fisher *et al.*, 1985; Yang *et al.*, 1987) has been controversial in operable breast cancer patients. As an unanimous principle, AX should be dissected in clinically AX-positive patients. However, the need for immediate AX dissection in clinically AX-negative patients has been questioned (Cascinelli *et al.*, 1987), because immediate AX dissection did not show any superiority in terms of survival compared to AX dissection performed only when clinical AX metastases appeared in a randomised clinical study (Fisher *et al.*, 1985). The main reason for AX dissection in clinically AX-negative patients is the staging of breast cancer, but the chances that AX are involved is only of the order of 20–25% in those patients (Noguchi *et al.*, 1991). On the other hand, it has also been reported that information about the presence or absence of IM metastases is important for estimating the prognosis of breast cancer patients (Veronesi *et al.*, 1985; Noguchi *et al.*, 1991). Nevertheless, routine IM dissection has not been justified as a staging procedure, because IM are involved only in 20% of the patients with operable breast cancer. If AX and/or IM metastases can be accurately estimated by biological parameters, then, dissection of AX and/or IM can be avoided in some breast cancer patients.

Leathem *et al.* (1984, 1985, 1987) found that lectin from the albumin gland of the Roman snail, *Helix pomatia* agglutinin (HPA), which recognises N-acetyl-galactosaminyl residues, binds to a population of breast-cancer cells associated with AX metastases. Other investigators (Fenlon *et al.*, 1987; Alam *et al.*, 1990; Brooks & Leathem, 1991) also reported that the presence of HPA binding correlated with AX metastases, whereas it has also been reported (Fukutomi *et al.*, 1989; Galea *et al.*, 1991) that HPA staining was not related to AX metastases. To our knowledge, however, none has investigated a relationship between HPA binding to primary tumour and the presence of IM metastases. The precentage of stainers (45%) and non-stainers (55%) are similar to the study of Fukutomi *et al.* (1989) but not in agreement with the one of Brooks *et al.* (1991), in which the incidence of PHA staining is 80% and PHA sensitivity with regard to lymph node status is 96%. The present study showed a significant correlation between HPA staining and AX or IM metastases. Expression of HPA binding site in breast cancer tissue may reflect the ability of a tumour to invade and metastasise. Brooks *et al.* (1991) suggested that HPA binding might provide valuable information in breast cancer patients in whom AX sampling has not been performed. In the present study, the predictive value of AX or IM metastases by HPA staining was equivalent with that by the discriminant function with clinical variables. HPA staining may provide an adjuvant to discriminant function with clinical variables. The difference in technique and lectin source between the works of Brooks *et al.* (1991) and our may have some bearing on the significance of the HPA staining, although the incidence of HPA staining (45%) was similar with the incidence of AX metastases (46%) in this study. Since it is troublesome for clinicians to calculate the discriminant function for each patient, and one of the principal factors included in the discriminant function was clinical axillary status, however, the usefulness of the discriminant function would be limited. HPA staining would be a more realistic alternative in the clinical setting.

Nevertheless, either HPA staining or the discriminant function was shown to be inaccurate with an accuracy of less than 80%. They are still far from satisfactory. In this study, it was confirmed that the histological status of AX and IM can provide important prognostic information in breast cancer patients. Therefore, it was concluded that AX dissection and biopsy of IM are important in the detection of regional lymph node metastases (Fentiman *et al.*, 1991; Noguchi *et al.*, 1991).

We express our thanks to R. Yashiki and N. Takamura for their excellent technical assistance.

### References

ALAM, S.M., WHITFORD, P., CUSHLEY, W., GEORGE, W.D. & CAMPBELL, A. (1990). Flow cytometric analysis of cell surface carbohydrates in mestatatic human breast cancer. *Br. J. Cancer*, **62**, 238–242.

ALTEVOGT, P., FOGEL, M., CHEINGSONG-POTOV, R., DENNIS, J., ROBINSON, P. & SCHIRRMACHER, V. (1983). Different patterns of lectin binding and cell surface sialytation detected on related high- and low-mestatatic tumor lines. *Cancer Res.*, **43**, 5138–5144.

BROOKS, S.A. & LEATHEM, A.J.C. (1991). Prediction of lymph node involvement in breast cancer by detection of altered glycosylation in the primary tumour. *Lancet*, **339**, 71–74.

CASCINELLI, N., GRECO, M., BUFALINO, R., CLEMENTE, C., GALLUZZO, D., DONNE, V.D., LELLIS, R.D., SACCHINI, V. & VERONESI, U. (1987). Prognosis of breast cancer with axillary node metastases after surgical treatment only. *Eur. J. Cancer Clin. Oncol.*, **23**, 795–799.

FENLON, S., BELL, E.J., ELSTON, C.W. & BLAMEY, R.W. (1987). Helix pomatia and ulex europeus lectin binding in human breast carcinoma. *J. Pathol.*, **152**, 169–176.

FENTIMAN, J.S. & MANSEL, R.E. (1991). The axilla: not a no-go zone. *Lancet*, **337**, 221–223.

FISHER, B., REDMOND, C., FISHER, E.R., BAUER, M., WOLMARK, N., WICKERHAM, L., DEUTSCH, M., MONTAGUE, E., MARGOLESE, R. & FOSTER, R. (1985). Ten-year results of randomized clinical trial comparing radical mastectomy and total mastectomy with or without radiation. *N. Engl. J. Med.*, **312**, 674–681.

FUKUTOMI, T., ITABASHI, M., TSUGANE, S., YAMAMOTO, H., NANASAWA, T. & HIROTA, T. (1989). Prognostic contributions of Helix pomatia and carcinoembryonic antigen staining using histochemical techniques in breast carcinomas. *Jpn. J. Clin. Oncol.*, **19**, 127–134.

GALEA, M.H., ELLIS, I.O., BELL, J., ELSTON, C.W. & BLAMEY, R.W. (1991). Prediction of lymph node involvement in breast cancer. *Lancet*, **338**, 392–393.

KAPLAN, E.L. & MEIER, P. (1958). Non-parametric estimation from incomplete observation. *J. Am. Stat. Assoc.*, **53**, 457–481.

KINNE, D.W. (1983). Surgical management of primary breast cancer. *Cancer*, **51**, 2540–2546.

LACOUR, J., LE, G.M., HILL, C., KRAMAR, A., CONTESSO, G. & SARRAZIN, D. (1987). Is it useful to remove internal mammary nodes in operable breast cancer? *Eur. J. Surg. Oncol.*, **13**, 309–314.

LEATHEM, A., DOKAL, I. & ATKINS, N. (1984). Carbohydrate expression in breast cancer as an early indicator of metastatic potential. *J. Pathol.*, **142**, A32.

LEATHEM, A., ATKINS, N. & EISEN, T. (1985). Breast cancer metastasis, survival and carbohydrate expression associated with lectin binding. *J. Pathol.*, **145**, 73A.

LEATHEM, A.J. & BROOKS, S.A. (1987). Predictive value of lectin binding on breast-cancer recurrence and survival. *Lancet*, **i**, 1054–1056.

MEJER, P., FERGUSSON, D.J. & KARRISON, T. (1989). A controlled trial of extended radical mastectomy. Ten-year results. *Cancer*, **63**, 188–195.

NOGUCHI, M., OHTA, N., KOYASAKI, N., TANIYA, T., MIYAZAKI, I. & MIZUKAI, Y. (1991). Reappraisal of internal mammary node metastases as a prognostic factor in patients with breast cancer. *Cancer*, **68**, 1918–1925.

STECK, R.A. & NICOLSON, G.L. (1983). Cell surface glycoprotein of 13762 NF mammary adenocarcinoma clones of differing metastatic potentials. *Exp. Cell Res.*, **147**, 255–267.

UNION INTERNATIONALE CONTRE LE CANCER (1987). Breast (ICD-0 174). In *TNM Classification of Malignant Tumours*, ed. 4, Harmer, M.H. (ed.) pp. 93–99. Springer-Verlag: Berlin.

VERONESI, U. & VALAGUSSA, P. (1981). Inefficiency of internal mammary nodes dissection in breast cancer surgery. *Cancer*, **47**, 170–175.

VERONESI, U., CASCINELLI, N., GRECO, M., BUFALINO, R., MORABITO, A., GALLUZZO, D., CONTI, R., LELLIS, R.D., DONNE, V.D., PIOTTI, P., SACCHINI, V., CLEMENTE, C. & SALVADORI, B. (1985). Prognosis of breast cancer patients after mastectomy and dissection of internal mammary nodes. *Ann. Surg.*, **20**, 702–707.

YANG, J.H., SLACK, N.H. & NEMOTO, T. (1987). Effect of axillary nodal status of the long-term survival following mastectomy for breast carcinoma: nodal metastases may not always suggest systemic disease. *J. Surg. Oncol.*, **36**, 243–248.